# The Proteolytic Value of Malt1Read before the Chicago Medical Society.

**Published:** 1899-03

**Authors:** Charles H. Miller

**Affiliations:** Instructor in physiological chemistry, Northwestern University Medical School


### Selection.

### THE PROTEOLYTIC VALUE OF MALT.[1]

By CHARLES H. MILLER, Ph. G., M. D.,
Instructor in physiological chemistry, Northwestern University Medical School.

(*From the Chicago Medical Record.*)

THE amylolytic or starch digesting power of malt, or properly prepared extracts thereof, has long been known, and the fact utilised in medicine. Its value in this regard is so marked and its

1. Read before the Chicago Medical Society.

action so evident that the presence of any other enzyme has, until recently, been overlooked. Attention has been attracted to the question of proteolytic power in malt by statements published by the manufacturers of an infant food (Milkine), wherein such value was claimed for it.

On being asked regarding the accuracy of such statements, I was not quite sure of the proper answer, and this paper is the result of trying to secure accurate information on the subject. Turning to the recent literature, it was found that writers on physiological chemistry do speak quite uniformly of an enzyme, unorganised in form, proteid in composition and proteolytic in function, capable of converting notable quantities of proteid matter into soluble, diffused peptones. Briefly reviewing standard authorities, we find that at first such belief was somewhat guardedly expressed.

Thorpe[2] says: "It seems very probable that diastase exercises a very marked influence on the insoluble albuminoids during the process of germination, converting them into soluble forms, and during the process of mashing it acts still further on these soluble albumins, converting them into those other nitrogenous substances which are not coagulated by heat."

Somewhat later, Richter,[3] 1892, in referring to albuminoids of vegetable origin, states: "When seeds sprout, the fibrin is converted into the soluble ferment called diastase. The other unformed ferments appear to be modified albuminoids."

Sadtler,[4] 1895, more precisely says: "Besides the diastase, a second soluble ferment is formed during the malting-process, the so-called peptase, which in the mash process changes the proteids of the malt into peptones and parapeptones." He is there quoting O'Sullivan."

Wright[5], 1897, an English authority on ferments and fermenting processes, says: "Another of the so-called soluble ferments or unorganised ferments present in malt is peptase, which, though brought into being during the malting process, lies mainly if not entirely dormant until suitable conditions, such as the mashtun, stimulate it into activity. Its function is to convert the unaltered proteid bodies or albuminoids into peptones, which are apparently of the highest importance in nourishing the yeast plant, owing to their great diffusibility." There seems to be a fair consensus of opinion that:

1. Peptase exists in the germinated, but not in the ungerminated barley.

2. Its most favorable medium is an acid one, *e. g.*, 2 per cent. lactic acid, in this respect resembling the pepsin of the gastric juice, whence its name was suggested.

3. High temperatures are fatal to it.

Thausing affirms kiln-dried malt to be more energetic than air-dried. In his table of ferments, organised and unorganised, Wright places peptase along with pepsin and trypsin, as those composing the proteolytic division "whose function is to change proteids into easily diffusible peptones."

Wiley[6], 1897, chief chemist, United States government, says: "In sprouting plants there appears to be a widely diffused ferment, capable of converting the proteids of the cotyledons into peptonoid bodies, thus fitting them for entering the tissues of the new plant."

When the perpetuation of the species is the object in view, nature is ever prodigal, hence, reasoning by analogy, we easily arrive at the conclusion that, if there is enough peptase to convert the legumin into peptone for the plant, it is reasonable to suppose that there is doubtless an enormous excess produced over the actual amount required, which would be available for converting other nitrogenous matter into similar peptones; and since vegetable and animal proteids are practically the same, this surplus power could be directed to the digestion of animal proteids as well as any other.

If this property is present to any extent in malt preparations its value dietetically and therapeutically is apparent and will be readily conceded. Its evident importance so impressed me that a line of experimentation was projected, the object of which was the study of this ferment peptase, and if possible to determine its proteolytic power, assuming all such value to reside therein. The work was completed as outlined, and the results are believed to be of sufficient value to record, being quite satisfactory.

Both coagulated egg-albumin and raw chopped beef, in amounts of five grams each, were subjected to the digestive action of five different malt preparations from the market—three semi-liquid and two dry—at a uniform temperature of 40° C., for six hours, in various media; acid, neutral and alkaline, and with uniform agitation. Control tubes, containing pepsin, and pancreatin in suitable media, and others, without any digestives, were worked through with the others. Eighteen tubes were required in each experiment to study the varying effect in different media. Thymolised water was utilised to prevent bacterial peptonisation.

The different media were prepared as follows :

Acid—2-10 per cent. hydrochloric acid (HCI).

2 per cent. lactic acid ($C_3H\ O_3$).

Alkaline—5-10 per cent. sodium carbonate ($Na_2CO_3$).

Neutral—Normal saline solution.

The amounts of digestives used were as follows :

Liquid malt extracts, 5-10 gram.

Dry diastase powders, 2-10 gram.

Dry pepsin, 5-100 gram.

Dry pancreatin, 1-10 gram.

*E.g.*, in each experiment, direct and for control; always acting on the stated amount of the proteid material. Large size test tubes were the vessels used, and in large water bath maintained at 40° C.

Briefly, the stages of digestion possible of recognition in artificial proteid digestion are :

A.—In acid media,

1. Acid albumin,
2. Albumose (anti),
3. Peptone.

B.—In alkaline media,

1. Alkali albumin,
2. Albumose (hemi).
3. Peptones.

The Nos. 1 are readily precipitated by neutralising the media, not by boiling. The Nos. 2 are readily precipitated by saturating the liquid with a neutral salt, as ammonia sulphate $(NH_4)_2SO_4$, or sodium sulphate ($Na_2SO_4$), and boiling. The Nos. 3, being soluble, are found in the filtrates after the above treatment, and can be recognised qualitatively by the biuret reaction, or approximately quantitatively by precipitating with tannic acid.

The method adopted to determine the extent of digestion was to weigh the undigested residue—after removing any excess of liquid—which, considering the relatively large amounts of material acted on, was grossly valuable; then I compared the amounts of all albumoses thrown down with sodium sulphate to saturation, and finally the tannic acid precipitates from the above filtrates were compared. Using the well-digested liquids from the pepsin and pancreatin tubes for marked results, and the liquids from the absent-ferment tubes for negative purposes, by comparison it was found practicable to keep fair tab on the work.

The derived proteids were not weighed. Chemists and those who have worked with organic precipitates will readily appreciate the difficulties attending exact weighing of them. Probably errors introduced in their purification more than counterbalance any increased reliability of results. So it can be said the accuracy of the results would not be markedly enhanced in these processes by weighing the obtained precipitates.

After a sufficient number of repetitions to get concordant results, the following deductions have been made :

1. Properly prepared, light-colored, semi-liquid extracts of malt, possessing strong amylolytic power, have also positive proteolytic value, though decidedly less marked than the better known ferments, pepsin and trypsin.

2. The reaction of the media bears an important relation to the obtained results. This influence is believed to be as follows in sequence of greater activity :

2 per cent. lactic acid.

5-10 per cent. sodium carbonate.

6-10 per cent. sodium chloride (neutral).

2-10 per cent. hydrochloric acid.

Judging from these experiments, and in the proportions employed, the proteolytic power of malt extracts is about 5 per cent. that of pepsin or pancreatin.

4. The dry diastase preparations were not found to possess proteolytic power. The best results were obtained by a freshly prepared extract of malt, which I made just before use.

The practical results are simple, but of extended importance. Good malt extract is a valuable food, concentrated and immediately assimilable. It is capable of immediately converting enormous quantities of nondiffusible carbohydrate food starches into soluble dextrin or maltose forms, ready for the body's use. It is in the amounts commonly administered capable of largely converting the proteids of an ordinary meal into diffusible peptones.

It is distinctively a pleasant medicine, and one readily combined with practically all other remedies, often completely obscuring their disagreeable properties. That this is appreciated by our friends, the proprietary men, is shown by the ready-made combinations of malt and other remedies forced to our attention daily.

Malt extract is a first-class remedy, and the simplest rules of combination render it doubly satisfactory to use.

Regarding administration, it should be given just before or with the meal, and can always be advantageously combined with lactic acid to pleasant tartness, other medicines not interfering.

In the above experiments, maltine was one of the digestives used, its amylolytic and proteolytic power was found to be of exceptionally high order—practically answering to the highest converting strength obtained. A teaspoonful of maltine will effect nearly as much peptonisation, in proper media, as two grains of pepsin.

BIBLIOGRAPHY.

1. Thorpe's Dictionary of Applied Chemistry, 1891, Vol. I., page 358.
2. Richter's Organic Chemistry, 1892, page 358.
3. Sadtler's Handbook of Industrial Chemistry, 2d ed., page 187.
4. Wright's Handbook for Brewers, 1897, pages 3, 88, 323
5. Wiley—Principles and Practice of Agricultural Analysis, 1897. page 412.
6. Long's Physiological Chemistry, 1894, page 88.

---

### SUBSTITUTION.

THAT substitution is practised by many pharmacists there can be no doubt. Messrs. Fairchild Brothers & Foster deserve the thanks of the profession for the bold and energetic way in which they have followed up and published the names of those who have compounded prescriptions with other forms of pepsin when their essence was prescribed. We can, however, but consider the statement of the secretary of the Druggists' League for shorter hours as something of an exaggeration, that more than a hundred persons are killed each year in New York by the substitution of one drug for another in making up prescriptions. Some of these substitutions are made, he says, deliberately to increase profits, others are made by mistakes of overworked and half-asleep prescription clerks.

It certainly cannot be that this secretary would have us believe that substitutions which kill are made deliberately to increase profits! If there is even a tithe of truth in his statement, it would appear that something more radical is demanded than simply shortening the hours of drug clerks. We prefer to believe that the secretary was misquoted, or that he spoke thoughtlessly, for it stands to reason that his statement must be a gross exaggeration.—*The Brooklyn Medical Journal.*

